# New Insights into the Complex Relationship between Weight and Maturity of Burgundy Truffles (*Tuber aestivum*)

**DOI:** 10.1371/journal.pone.0170375

**Published:** 2017-01-26

**Authors:** Ulf Büntgen, István Bagi, Oszkár Fekete, Virginie Molinier, Martina Peter, Richard Splivallo, Maryam Vahdatzadeh, Franck Richard, Claude Murat, Willy Tegel, Ulrich Stobbe, Fernando Martínez-Peña, Ludger Sproll, Lisa Hülsmann, Daniel Nievergelt, Barbara Meier, Simon Egli

**Affiliations:** 1 Department of Geography, University of Cambridge, Cambridge, United Kingdom; 2 Swiss Federal Research Institute WSL, Birmensdorf, Switzerland; 3 CzechGlobe Research Institute CAS and Masaryk University Brno, Brno, Czech Republic; 4 Truffleminers Ltd, Taksony Kinizsi, Hungary; 5 Goethe University Frankfurt, Institute for Molecular Bio Science, Frankfurt, Germany; 6 Integrative Fungal Research Cluster (IPF), Frankfurt, Germany; 7 UMR 5175 CEFE - University of Montpellier, Montpellier, France; 8 Université de Lorraine, UMR1136 Interactions Arbres-Microorganismes, Vandoeuvre-lès-Nancy, France; 9 INRA, UMR1136 Interactions Arbres-Microorganismes, Champenoux, France; 10 Chair of Forest Growth, Albert-Ludwigs University, Freiburg, Germany; 11 Chair of Forest Botany, Albert-Ludwigs University, Freiburg & Deutsche Trüffelbäume, Radolfzell, Germany; 12 Agrifood Research and Technology Centre of Aragon CITA, Zaragoza, Spain; 13 European Mycological Institute EGTC-EMI, Soria, Spain; University of Perugia, ITALY

## Abstract

Despite an increasing demand for Burgundy truffles (*Tuber aestivum*), gaps remain in our understanding of the fungus’ overall lifecycle and ecology. Here, we compile evidence from three independent surveys in Hungary and Switzerland. First, we measured the weight and maturity of 2,656 *T*. *aestivum* fruit bodies from a three-day harvest in August 2014 in a highly productive orchard in Hungary. All specimens ranging between 2 and 755 g were almost evenly distributed through five maturation classes. Then, we measured the weight and maturity of another 4,795 *T*. *aestivum* fruit bodies harvested on four occasions between June and October 2015 in the same truffière. Again, different maturation stages occurred at varying fruit body size and during the entire fruiting season. Finally, the predominantly unrelated weight and maturity of 81 *T*. *aestivum* fruit bodies from four fruiting seasons between 2010 and 2013 in Switzerland confirmed the Hungarian results. The spatiotemporal coexistence of 7,532 small-ripe and large-unripe *T*. *aestivum*, which accumulate to ~182 kg, differs from species-specific associations between the size and ripeness that have been reported for other mushrooms. Although size-independent truffle maturation stages may possibly relate to the perpetual belowground environment, the role of mycelial connectivity, soil property, microclimatology, as well as other abiotic factors and a combination thereof, is still unclear. Despite its massive sample size and proof of concept, this study, together with existing literature, suggests consideration of a wider ecological and biogeographical range, as well as the complex symbiotic fungus-host interaction, to further illuminate the hidden development of belowground truffle fruit bodies.

## Introduction

Fruit bodies of the symbiotic Burgundy truffle (*T*. *aestivum* Vittad.) [1] range amongst the most expensive of gourmet foods [2]. *T*. *aestivum* can be found almost throughout the entire year and most of Europe [3–5]. Despite a long history of human consumption, there are still many open questions concerning the origin, biogeography and ecology of most truffle species [1–5], including *T*. *aestivum*. The ecological and commercial interest in this ectomycorrhizal ascomycete has rapidly increased over the past few years [5], primarily because of a rising demand for truffles worldwide [2, 6, 7], as well as possibly also due to some declining harvests of the Périgord truffle (*T*. *melanosporum* Vittad.) in parts of its Mediterranean distribution [8]. The wide ecological range of *T*. *aestivum* [5], its prolonged harvest season in comparison to *T*. *melanosporum*, as well as a much lower price level though higher cultivation potential [2], further contribute to the increasing commercial importance of *T*. *aestivum*.

An array of abiotic and biotic factors, such as climatic variation and symbiotic association, are important for the subsurface process of truffle fruit body formation [5]. Soil-borne microbial communities [9], for instance, have been reported to affect the development and functioning of ectomycorrhiza symbiosis and might also affect truffle production [10]. Changes in the total carbon budget of symbiotically associated host trees during a season have also been described to influence truffle growth [11–13]. This finding is well in agreement with an earlier fruiting season at the transition from spring to summer that geographically corresponds with generally warmer regions [14–16]. More autumn- and winter-oriented truffle fruiting is, however, mostly found at higher latitudes and/or altitudes.

Among many central European countries [3–5], Hungary comprises an ideal environment for the natural and cultivated growth of *T*. *aestivum* [17]. The most productive region, called Jászság, is situated between the Danube and Tisza rivers in the middle of the fertile Hungarian plain. Warm and dry summers are typical features for this agriculturally dominated flatland, which is characterized by fluvial alluvia comprising chernozems, fluvisols, solonchaks and arenosols.

Here, we aim at understanding if *T*. *aestivum* fruit bodies are first growing to a certain size and then maturing—as often reported for epigeous basidiocarps and ascomycetes—or if these lifecycle-inherent processes chronologically overlap, i.e. the size at which maturity is achieved varies. We compiled and analyzed three independent *T*. *aestivum* datasets that were generated in 2014 and 2015 in an orchard in Hungary, as well as between 2010 and 2013 in an orchard in Switzerland. While providing a timely literature review of biotic and abiotic factors possibly influencing truffle weight and maturity, we critically discuss our findings in a broader conceptual framework. Moreover, we define and recommend research priorities and agendas to gain further insights into the mysterious, subterranean truffle kingdom.

## Materials and Methods

**Fig 1:**
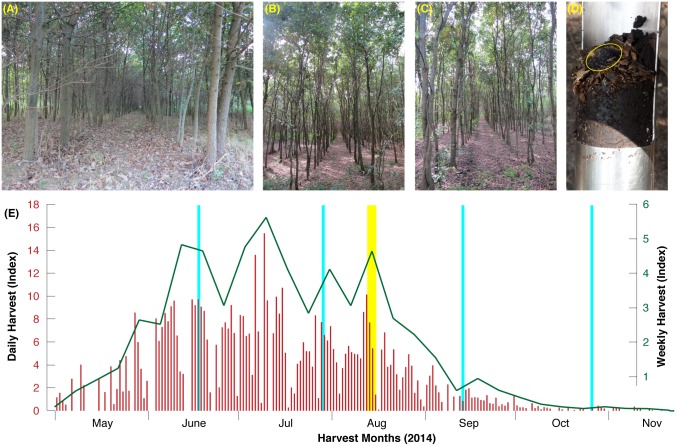
**(A-C)** The Jászivány orchard in central Hungary, which was naturally colonized by *T*. *aestivum* some years ago. Fruit bodies are generally harvested within the upper soil layer **(D)**, and between June and August **(E)**. The yellow vertical bar refers to the three sampling days of the 2014 campaign (Aug 12–14) that resulted in 2,656 fruit bodies, whereas the light blue vertical bars display the timing of the repeated 2015 measurements (June 16, July 28, September 12, and October 26) that resulted in 4,795 fruit bodies.

The majority of this study was conducted in a planted oak stand in central Hungary (Fig 1A–1C). Trees are 21-year old and cover 41.5 ha of flat terrain ~90 m asl in the county of Jász-Nagykun-Szolnok near the village Jászivány (around 47.5° N and 20.2° E). This plantation was naturally colonized by *T*. *aestivum* some years ago. The recent truffle orchard, called Jászivány, is characterized by a uniform distribution of *Calcic Chernozem* (Loamic) soil. Being poor in stone content, its texture corresponds to a silt-clay-loam (12% silt, 38% clay, 50% loam) with a high organic matter content and a high biological activity. The pH value of this very deep mull-like soil spatially varies from 6.9–8.2 (in water). The continental climate exhibits approximately 2000 sunshine hours per year, resulting in an annual temperature mean of 10.2 C°. Nevertheless, exceptionally cold winters are contrasted by warm summers. Average annual precipitation totals fluctuate between 560 and 580 mm, with seasonal hydroclimatic minima in summer.

The Jászivány orchard is inhabited by nearly 70% English oak and 30% Turkish oak (*Quercus robur* L. and *Q*. *cerris* L.). These trees were planted in 1994 on former black locust wood (*Robinia pseudoacacia* L.) that still accounts for ~1–5% of all trees. Oak height is 8–12 m and stand density ~4000 trees/ha, with an average distance of 1.0 m between the individual trees and 2.5 m between each tree row. Light thinning was performed in 2010 and 2012. According to our own phenological observations and high-resolution band dendrometer measurements, the vegetation period usually starts in early-April and lasts until the end of November. With an annual average of 4.3 tons during the last four years (varying from 2.3–6.1 tons between 2011 and 2014), the yearly fruit body production of *T*. *aestivum* is extremely high (~104 kg/ha). Although systematic truffle harvests with trained dogs begin in early-May and uninterruptedly continue until the end of November, the main fruiting peaks are distributed throughout mid-June to mid-August [17] (Fig 1E).

**Fig 2:**
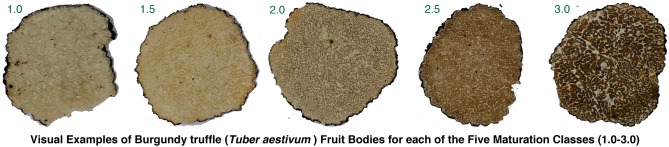
Thin sections of five individual *T*. *aestivum* fruit bodies from Hungary (2014 Aug 12–14), which provide a visual proof of concept that macro-morphological gleba characteristics (color and structure) can be used to distinguish the five maturation classes (1.0, 1.5, 2.0. 2.5 and 3.0) in the field.

After intense harvesting with trained dogs between 12 and 14 August, 2014, we recorded the weight (gram) and maturation of a total of 2,656 provisionally cleaned *T*. *aestivum* fruit bodies. We empirically defined five maturation classes (1.0, 1.5, 2.0. 2.5 and 3.0), which were visually distinguished in the field (Fig 2). These five classes separate the following fruit body types: Unripe with white and partly even transparent gleba (1.0), slightly ripe with white gleba (1.5), mainly ripe with beige-grey gleba (2.0), ripe with light brown gleba (2.5), and fully ripe with brown gleba (3.0). Overripe truffles were not considered by the hunters due to their insignificant economic value. All fruit bodies were double sliced at two opposing surface positions to best identify homogeneity of the gleba color and structure, and to ensure that the attributed maturation class represents most of the fruit body (see Fig 2 for the specific macro-morphological gleba characteristics). This assessment was independently performed by three experienced mycologists. While the advantage of this method is cost and labor efficiency, additional parameters for the firm description of the maturity/ripening relationship were not considered. Information on glucose level, melanin content, volatile composition, spore quantity and ornamentation, as well as related bacterial communities, for instance, which can be achieved via state-of-the-art Illumina Sequencing Technology, were traded for a large number of fresh samples and an easy workflow within the field. Belowground fruit bodies were collected during the second phase of the main fruiting period from August 12–14, 2014 (Fig 1E). Based on the optical examination of three scientists, our five-level maturation classification was designed to best matching previously introduced fruit body groupings based on much more accurate microscopic techniques [18].

To account for different periods throughout a truffle fruiting season and thus to account for intra-annual variability of fruit body weight and maturity, we performed four additional field surveys in 2015 in the same orchard in Hungary between mid-June and late-October. A total of 4,795 *T*. *aestivum* fruit bodies were harvested with trained dogs on 16 June (34.5 kg), 28 July (30.2 kg), 12 September (21.7 kg) and 26 October (4.9 kg). The weight (gram) and maturity class (1.0, 1.5, 2.0. 2.5 and 3.0) of each truffle was recorded.

To further increase our understanding of how the fruit body formation and maturation of *T*. *aestivum* may evolve at intra- and interannual time-scales, a total of 81 fruit bodies were collected between 2010 and 2013 within a Swiss orchard. This dataset represents a significant update from [19], and originates from artificially inoculated pine (*Pinus nigra* J.F. Arnold) and oak (*Quercus robur* L.) trees in southwestern Switzerland [19]. Samples were collected over four consecutive fruiting seasons between September 2010 and September 2013 (i.e. 10 Sep, 03 Oct, 30 Nov 2010, 18 Aug, 01 Nov, 22 Dec 2011, 21 Aug, 19 Dec 2012, 19 Sep 2013). Morphological spore observations together with PCR primers UNCI ⁄ UNCII were utilized for the identification of *T*. *aestivum* [19, 20]. Based on rigorous microscopic techniques, fruit body maturity of each of the 81 truffle fruit bodies from Switzerland was determined as the ratio between the number of asci containing melanized spores and the total number of asci [18]. Statistical analyses and significance levels were calculated in Excel:Mac2011 version (14.6.9), as well as using the R package multcomp [21, 22]. All figures were finalized via Adobe Illustrator CS6 (version 16.0.4). No specific permissions were required for any of the truffle sampling sites and campaigns in both, Hungary and Switzerland. Moreover, none of the field studies involved endangered or protected species.

## Results

**Fig 3:**
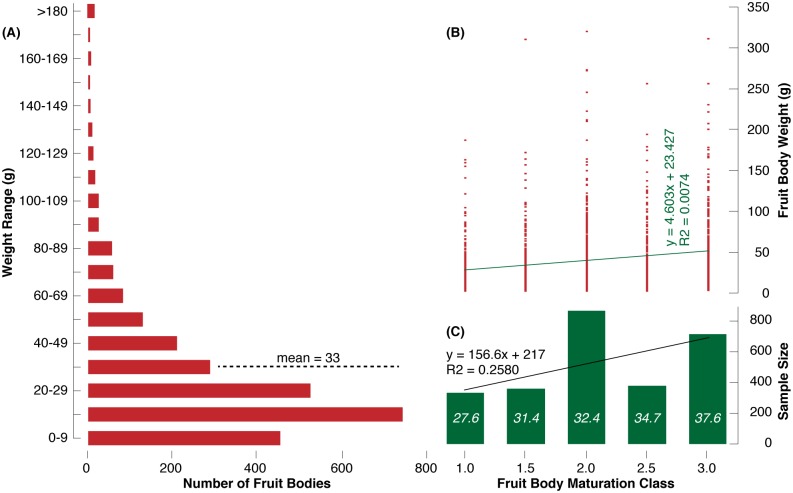
**(A)** Frequency distribution of the weight of all 2,656 *T*. *aestivum* fruit bodies from the 2014 Hungarian survey (Aug 12–14). **(B)** Relationship between the individual fruit body weight and the five maturation classes ranging from 1.0–3.0. Note that the record-breaking fruit body of 755 g has been excluded for scaling issues. **(C)** The number of fruit bodies (sample size) per maturation class with white numbers referring to the corresponding mean weight (g).

Fresh weight of the 2,656 individual *T*. *aestivum* fruit bodies from the August 12–14, 2014 harvest in Hungary varied from 2–755 g with an average of 33 g (Fig 3A). A total of 1,699 fruit bodies weighted <30 g, 818 samples ranged from 30–89 g, and the remaining 139 truffles weighted >90 g. Although the giant of 755 g clearly marks an exception, a better understanding of the ecological and climatological conditions, as well as the time needed for such a fruit body to grow, is more than scientific curiosity alone [23]. Fruit bodies of different weight were found in all five maturation classes (Fig 3B). There is a slight positive trend between mean fruit body weight and maturation class (Fig 3C). At the same time, there also persists a high level of variability since small and large truffles can be more or less mature.

**Fig 4:**
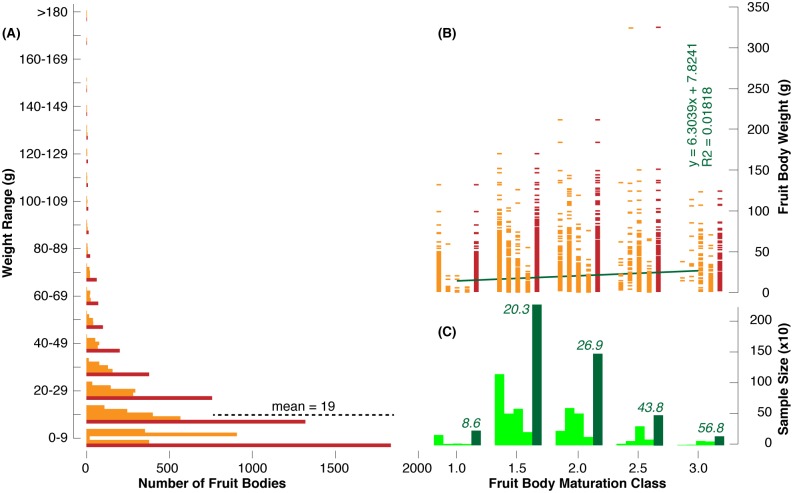
**(A)** Frequency distribution of the weight of all 4,795 *T*. *aestivum* fruit bodies (dark red) from the 2015 Hungarian survey, with results of the four individual Jun-Oct sampling campaigns being highlighted in orange. **(B)** Relationship between the individual fruit body weight and the five maturation classes (1.0–3.0) of all (dark red) and the monthly samples (orange), as well as **(C)** the number of fruit bodies (sample size) per maturation class with dark green numbers referring to the corresponding mean weight of all fruit bodies per class (g).

**Fig 5:**
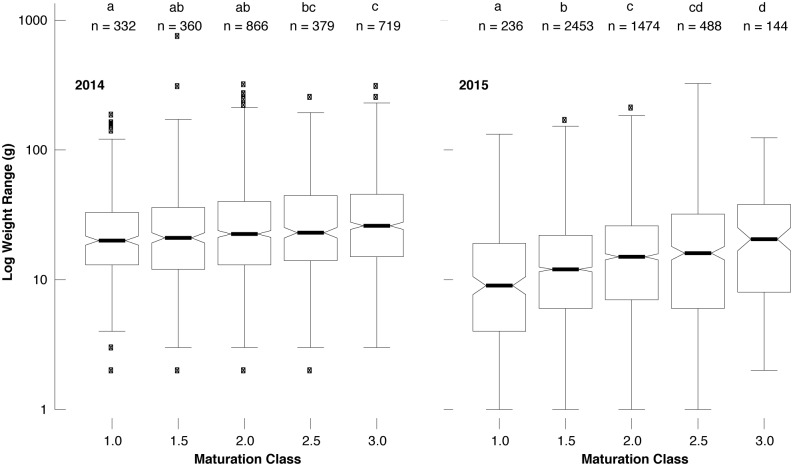
Boxplots and Tukey HSD post-hoc test showing the differences of the individual truffle weights (gram per fruit body at logarithmic scale) between the five maturation classes (1.0, 1.5, 2.0, 2.5, 3.0) in 2014 and 2015 (left and right side). Within each year, maturation classes that do not share the same letter (upper x-axis) have significantly different means in pairwise comparison. Box plots contain several information: the box-length covers the inter-quartile range of the data, the median is marked with a bold line, and notches define an approximate confidence interval of the median, with the whiskers including 99.3% of the data if normally distributed.

**Fig 6:**
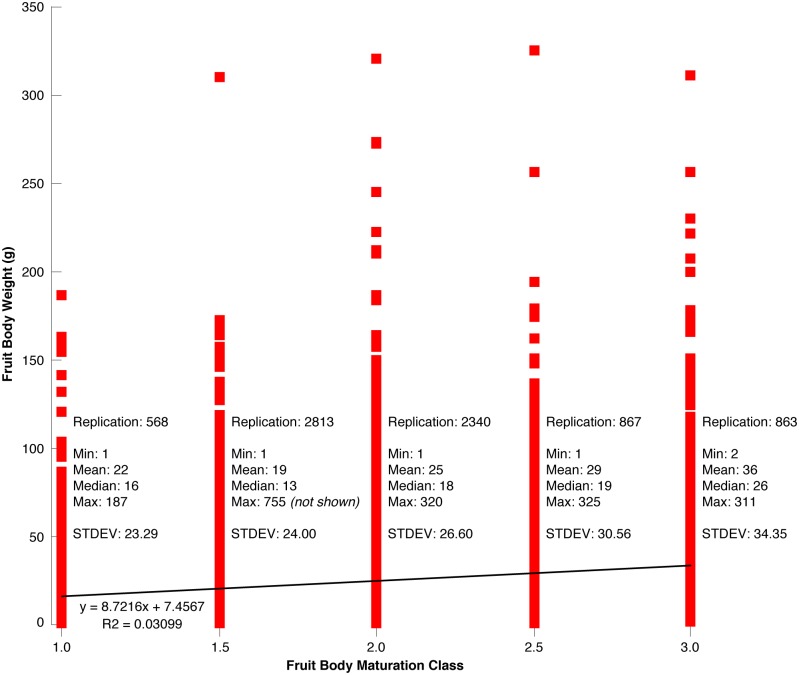
Relationship between the weight (g) and maturity (%) of a total 7,451 *T*. *aestivum* fruit bodies (~180 kg) that were collected throughout two growing seasons in 2014 and 2015 in an orchard in central Hungary.

During the four consecutive Hungarian field surveys throughout the 2015 truffle season, we recorded the weight and maturity level of 4,795 individual *T*. *aestivum* fruit bodies in June, July, September and October. Their fresh weight substantially varied below 325 g with an average of 19 g (Fig 4A). A total of 3,904 fruit bodies weighted <30 g, 829 samples ranged from 30–89 g, and the remaining 62 truffles weighted >90 g. Again, fruit bodies of different weight were found in all five maturation classes (Fig 4B). Although there is a small positive trend between mean fruit body weight and maturation class (Fig 4C), there also persists a high level of variability since small and large truffles can be more or less mature. The four intra-annual sampling campaigns revealed quite comparable results: Weight and maturity of *T*. *aestivum* fruit bodies is mainly unrelated. Nevertheless, Tukey HSD post-hoc tests between the log-transformed weights of different maturation classes calculated separately per year revealed, almost exclusively, statistically significant differences between the maturation classes while the weight consistently increased with maturation (Fig 5). The joint assessment of all 7,532 *T*. *aestivum* fruit bodies from Hungary exhibited strong evidence of the coexistence of small-ripe and large-unripe individuals (Fig 6).

**Fig 7:**
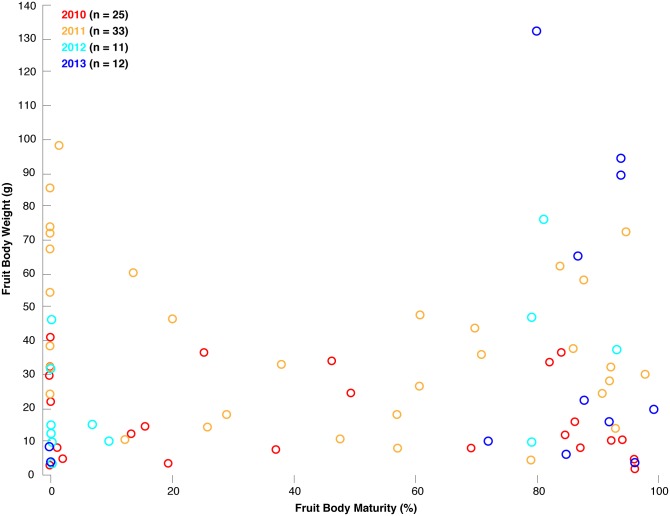
Relationship between fruit body weight (g) and maturity (%) of 81 *T*. *aestivum* that were collected throughout the four growing seasons between 2010 and 2013 in an orchard in southwestern Switzerland [19].

A recently updated study over four *T*. *aestivum* fruiting seasons in Switzerland between 2010 and 2013 [19] confirmed our results from Hungary. The weight of 81 *T*. *aestivum* fruit bodies from Switzerland significantly varied from 1 to 132 g, with an average of 32 g. Ranging from 0–99%, the mean maturity level of all 81 truffles is 47%. Although the annual harvests fluctuated considerably (2010: 25 truffles, average weight: 18 g ±13 (STD); 2011: 33 truffles, 43 g ±25; 2012: 11 truffles, 27 g ±25; 2013: 12 truffles, 39 g ±25), no relationship was found between fruit body weight and maturity (Fig 7). Microscopic evaluation confirms that specimens, independent of their size, can be fully immature (0% ascii with melanized spores) or fully mature (99%).

## Discussion

### Open research questions

Although recent work addressed the taxonomic status of *T*. *aestivum* [20, 23–26], as well as its aroma profile [19, 27–29], cultivation potential [5, 30–32], and soil requirements [33–35], much of its lifecycle and autecology is still poorly understood. At the same time, destructive subterranean sampling campaigns under natural (wildlife) and artificial (planted) conditions is either inefficient or prohibited, and at best only provides a limited glimpse in time.

Species-specific differences in the length of truffle fruiting seasons raise questions about growth periodicity. While one or more lifecycles per season with primordia formation between spring and autumn followed by maturation are discussed for *T*. *melanosporum* [3, 36], no such information is yet available for *T*. *aestivum* [5]. In contrast to other truffle species that have a relatively short ascocarp-producing season, the extended fruiting period of *T*. *aestivum* between early-June and March in the succeeding year implies several generations of shorter lifecycles [3, 4].

Unraveling the relationships between the biological process of fruit body maturation and the development of truffle aroma is another pending task. While maturation probably affects the aroma of the white truffle *T*. *borchii* [18], it is at best marginally important for the aroma of *T*. *aestivum* [19, 29]. Moreover, aroma formation is subject to microbes inhabiting fruit bodies [37–39], which suggests different factors that might act together in prompting truffle aroma, and eventually in determining the temporal development of quality. Furthermore, the commercial truffle market not only considers the size but also the shape of fruit bodies as valuable criteria. Since specimens are classified according to their flavor and dimension, large ascocarps with regular shape usually receive a higher price per gram. Also under scrutiny, it appears most likely that the complexity of truffle maturation and ripening is associated with at least three different traits: Darkening of gleba as a result of the biological maturation and pigmentation of spores and an increase in melanin content [40]. Selection of and interaction with dynamic bacterial and yeast communities [41] that might be involved in the maturation and ripening process of ascocarps [42], as well as in aroma production [37–39]. Increasing carbohydrate content, with glucose as the main component, reaches four times higher values in fully mature *T*. *melanosporum* ascocarps (up to 30% of the dry weight) in comparison with unripe ascocarps [40]. If and how these traits temporally coincide and how they are directly or indirectly linked with biotic and abiotic drivers is, however, still unclear.

### New evidence and remaining uncertainties

Our study provides answers to some of the above questions. The spatiotemporal coexistence of small-ripe and large-unripe *T*. *aestivum* specimens in Hungary and Switzerland—in consecutive years as well as within one season—suggests that truffle maturation can happen independently of fruit body formation. The lifecycle of every single fruit body is probably much less dependent of seasonal, environmental triggers than previously assumed. In fact, there is no clearly defined optimal time for primordia development, growth and maturation, but rather a constant and parallel cycle of all processes. This finding stresses the putative importance of other ecological factors such as mycelial connectivity, soil chemical and physical parameters as well as microclimatology for truffle growth or the pace of ripening. All 7,532 fruit bodies from Hungary developed under comparable soil conditions within the uppermost 10 cm of agricultural ground (Fig 1D). This finding is well in agreement with the long-term experience gained by local truffle hunters. Although an estimated 30–40 tons of fresh *T*. *aestivum* as harvested during the last ten years, it is still unclear if (and if yes how) size, ripeness, shape and peridium texture are linked to each other and/or how such traits are (if at all) affected by abiotic factors. Furthermore, the role of (micro) climate and the hunting preference of dogs still remain poorly understood. Concerning ecological site homogeneity, the regularly planted oaks (Fig 1A–1C) probably contribute to a comparable microclimate with little differences in soil humidity and chemistry.

Evidence from repeated field surveys in Hungary and its confirmation from a recently updated Swiss long-term study proposes that the growth and maturation of *T*. *aestivum* fruit bodies often occurs independently. In contrast to the assumption that big truffles also tend to be ripe, the spatiotemporal coexistence of small-ripe and large-unripe *T*. *aestivum* fruit bodies is indicative of a rather heterogeneous size-ripeness relationship. This pattern has not been previously reported, neither from observations under regular field nor laboratory conditions [4, 5], because systematic sampling campaigns were missing and *T*. *aestivum* fruit bodies have not yet been obtained successfully in laboratories. Our results thus demonstrate that the size/weight-maturity relationship for some hypogeous fungi can differ from the fact that species-specific final sizes sometimes exist for mature epigeous basidiomycetes and ascomycetes [43, 44].

Nevertheless, at least four aspects of our study remain poorly understood and thus call for critical discussion: i) How representative are our independent findings from the three different surveys in Hungary and Switzerland, ii) how well is the degree of fruit body maturation and ripening perceived in the light of our simple field assessment, iii) how selective appears the dog-based fruit body hunting, and iv) how old are the harvested fruit bodies?

i) Limitations not only arise from the specific biogeographic conditions of the Hungarian orchard that is certainly not representative for all other wild and cultivated *T*. *aestivum* habitats in Europe. Some further restrictions might also derive from the short sampling intervals that disregards most of the harvest season. Nonetheless, our four individual samplings were performed within different productivity phases of the entire fruiting period that extends from May-November, with the main production peak(s) falling into the June-August high-summer season (Fig 1E). Further uncertainty may originate from our simplistic field-based maturation classification that does not apply microscopy to distinguish between empty asci or the absence of asci, mature asci with immature spores (hyaline color and without or incomplete formed ornamentation) and asci with mature spores (yellow-brown color with a fully formed ornamentation) [18]. The Swiss long-term study [19] provides independent evidence for comparison with the two Hungarian surveys. It appears supplementary as it covers not only different biogeographical and seasonal aspects but also spans four consecutive seasons of systematic truffle harvest from 2010–2013.

ii) The spatiotemporal coexistence of similarly sized mature and immature fruit bodies raises questions concerning possible drivers of fruit body formation, including growth and maturation. The process of ripening, the production of volatiles, the determination of final ascocarp sizes at maturity, and the role of abiotic as well as biotic factors are all not understood. This level of uncertainty includes traits of the fruit body-forming vegetative mycelia such as size and age, as well as the ecophysiological and nutritional status of the host plants. It is also still under scrutiny if maturation and/or ripening is genetically programmed to proceed in a certain period of time after compatible mating strains fusion, and if growth is mainly driven by climatic, edaphic and symbiotic interaction. Complete fruit body formation, as well as maturation of heterothallic fungi that have a single MAT locus with two idiomorphs [45], depend on the fusion of two individuals bearing opposite mating types. While both mating types are necessary for maturation because ascospore formation needs sexual crossing, it remains puzzling how this is achieved by different truffle species (see [46] for *T*. *magnatum* and [47–49] for *T*. *melanosporum*). It is also debated whether two undifferentiated hyphae or hyphae that formed sexual reproductive organs fuse, and/or if hyphae are fertilized by mitospores that function as spermatia [50].

Unraveling the role that biochemical differences between more or less mature fruit bodies play in determining gastronomic value also remains an open task. The spore-based melanin content (up to 15% by dry weight in *T*. *melanosporum*) can be used as a biomarker of the degree of ascocarp development and of attainment of maturity [40]. For example, anandamide (a stimulant of melanin synthesis) is not detectable during the early maturation stage of *T*. *melanosporum* [51]. Different meanings of maturation (e.g. the biological maturation of spores) and ripening (e.g. the flavor production for animal attraction) add further ambiguity. Maturation refers to the biochemical process of spore maturation that goes along with increasing melanin content, whereas ripening denotes the flavor production [52]. The subjective consideration of odor also remains challenging as it may differ within and between animals and humans. Species-specific variability in the composition of volatiles is not only related to the fungus’ development stage and maturation [18], but also depends on genotypes [19], as well as the influence of external abiotic and biotic factors. In addition, such changes can qualitatively and quantitatively vary among ascocarps of the same truffle species and even the same individual [19], as well as within and between host species. A multifactorial approach to better define truffle maturity should therefore consider gleba color, spore maturity as revealed by the number of spores present in asci and their ornamentation, aromatic and chemical composition and bacterial communities. It should be further noted that humans only perceive a small fraction of the total variety of volatiles [28, 53]. Not more than six active aromatic compounds have been described for *T*. *aestivum* from northeastern Spain, including sulfur, alcohols and ketones [28], and it has been argued that the concentration of single aroma constituents can greatly vary between fruit bodies from the same species and site [19]. Variability in the concentration of eight carbon-containing volatiles (C8-VOC) in *T*. *aestivum*, which are characterized by truffle smell [54], has been linked to a specific genotype [19, 29]. Unlike evidence from other truffle species, geographic origin and stage of maturity seem to have only minor effects on the aroma profile of *T*. *aestivum* itself [19, 29].

iii) Dogs probably do not base their choice on chemical signals primarily related to maturity stages and/or odors perceived by humans. Although the intensively trained dogs often provide unique access to a large gradient of fruit body maturity, there are still many open questions related to their hunting behavior. Additional uncertainty arises from our limited understanding of their key-volatiles, and further complexity originates from breed variety. Our results from Hungary and Switzerland suggest that dogs most likely harvest independent of truffle maturation stages. The maturity level seems to be either unimportant for the chemical signal perceived by dogs and/or dogs do not distinguish in the soil between different odors perceived by humans after harvesting. Experimental confirmation is, however, still missing, and researchers should distinguish between the initial signal perceived by dogs through the soil and the subsequent smell of harvested truffles. The high amount of immature fruit bodies harvested in Hungary and Switzerland suggests that dogs and maybe also other mammals are not only (or at least firstly) attracted by ripe specimens. The wide range of simultaneously collected ripe and unripe fruit bodies further indicates that our study is likely not biased by dog-specific hunting preferences. Nevertheless, caution is advised since other non-carnivorous mammals such as mice and squirrels may use truffles as principal food and therefore possibly select for glucose content. More insight is also needed for disentangling the biological meaning of the truffles’ aroma production as an attractant to mature fruit bodies. Other attributes such as the content of glucose to select for being eaten or not might be important as well, particularly for animals that use them as principal food and which are the main vectors for distribution.

iv) It is unknown how long individual fruit bodies are hidden in the soil before they are harvested. Moreover, it is unclear if the odor of *T*. *aestivum* fruit bodies is changing during their formation and whether they have no attractive odor prior to their ultimate harvesting, because no correlation between maturity stage and odor was found [19, 29]. Similar to previous work [42], our study represents subsets of fruit bodies that were harvested within limited time, with an unknown number of undetected truffles. Although still a compromise and typically prohibited in economically designed orchards, we advocate for *in situ* excavations to gain further insight into the subterranean lifecycle of truffles. Likewise, reliable information on fruit body decay is scarce, as fungal decomposition depends upon the species involved and can range from a few days to several years [55]. Based on our own experiences and due to their melanized peridium, truffle fruit bodies would rather belong to the slowly decomposing species. Several factors that regulate the production and turnover of ectomycorrhiza tissues, including mycelium, and its role in soil carbon dynamics have been recently discussed [56]. Among others, the melanin content has been proposed as a possible determinant of decomposability [57].

## Conclusions

This study demonstrates that 7,451 *T*. *aestivum* fruit bodies from Hungary (~180 kg), harvested by trained dogs during two years and different periods in a very productive truffle orchard, were distributed throughout five maturation classes. Our results supplement the long-term observations of local truffle hunters: Although there is a slight positive trend between mean fruit body weight and maturation class, small truffles can be ripe while large truffles can be unripe. In addition, we found no evidence for a systematic link to one (predominant) or more (interacting) external factors. An independent long-term assessment of another 81 *T*. *aestivum* specimens from four harvesting seasons between 2010 and 2013 in Switzerland (~2 kg) also disproves any statistically robust relationship between fruit body weight and maturity. The predominant co-occurrence of small-ripe and large-unripe *T*. *aestivum* fruit bodies is, indirectly, also indicative of a rather heterogeneous size-ripeness association, which generally differs from a more or less species-specific final size of some mature epigeous basidiomycetes and ascomycetes. This circumstance might be related to the subterranean mode of truffle life with specific growth conditions and dependencies on the surrounding ecology, including soil conditions and microclimatic variations, as well as effects of microbial communities and mycelial connectivity.

## Supporting Information

S1 Table This excel-file contains all data relevant to our manuscript. (XLSX)
